# HMBA Enhances Prostratin-Induced Activation of Latent HIV-1 via Suppressing the Expression of Negative Feedback Regulator A20/TNFAIP3 in NF-*κ*B Signaling

**DOI:** 10.1155/2016/5173205

**Published:** 2016-07-27

**Authors:** Duchu Chen, Huiping Wang, Jude Juventus Aweya, Yanheng Chen, Meihua Chen, Xiaomeng Wu, Xiaonan Chen, Jing Lu, Ruichuan Chen, Min Liu

**Affiliations:** ^1^State Key Laboratory of Stress Cell Biology, School of Life Sciences, Xiamen University, Xiamen, Fujian 361005, China; ^2^Department of Genetics, Xuzhou Medical College, Xuzhou, Jiangsu 221000, China

## Abstract

In the past decade, much emphasis has been put on the transcriptional activation of HIV-1, which is proposed as a promised strategy for eradicating latent HIV-1 provirus. Two drugs, prostratin and hexamethylene bisacetamide (HMBA), have shown potent effects as inducers for releasing HIV-1 latency when used alone or in combination, although their cellular target(s) are currently not well understood, especially under drug combination. Here, we have shown that HMBA and prostratin synergistically release HIV-1 latency via different mechanisms. While prostratin strongly stimulates HMBA-induced HIV-1 transcription via improved P-TEFb activation, HMBA is capable of boosting NF-*κ*B-dependent transcription initiation by suppressing prostratin-induced expression of the deubiquitinase A20, a negative feedback regulator in the NF-*κ*B signaling pathway. In addition, HMBA was able to increase prostratin-induced phosphorylation and degradation of NF-*κ*B inhibitor I*κ*B*α*, thereby enhancing and prolonging prostratin-induced nuclear translocation of NF-*κ*B, a prerequisite for stimulation of transcription initiation. Thus, by blocking the negative feedback circuit, HMBA functions as a signaling enhancer of the NF-*κ*B signaling pathway.

## 1. Introduction

The highly active antiretroviral therapy (HAART) can significantly reduce the plasma viral loads of individuals infected with human immunodeficiency virus (HIV-1) to undetectable levels, thereby slowing down the clinical progression to full-blown AIDS (acquired immune deficiency syndrome). In spite of this fact, long-term treatment with HAART fails to totally eradicate the virus in infected individuals due to the persistence of a reservoir of resting CD4+ T cells which harbor transcriptionally dormant but replicative-competent proviruses. This reservoir of HIV-1 is a permanent source for the rebound in viral load upon interruption of HAART. Thus, in the last couple of years, great emphasis has been put on discovering and employing strategies aimed at reducing the size of the latent HIV-1 reservoir by reactivating transcription of the latent provirus. It is believed that such strategies would not only permit the killing of these latently infected cells by the viral cytopathic effects or their elimination by the host immune system following viral reactivation, but also complement HAART to prevent the spread of the infection by the nascent virus [1–4].

Synonymous with transcription of messenger RNA (mRNA) from protein-coding genes in host cells, the transcription of the HIV-1 genome from integrated provirus is also performed by RNA polymerase II (RNAPII) in a process consisting of several highly coordinated stages [5, 6]. While it is a known fact that NF-*κ*B-dependent transcriptional activation is critical for the preinitiation/initiation of HIV-1 proviruses, the recruitment of P-TEFb (positive transcription elongation factor b) by Brd4 (bromodomain containing protein 4) during the elongation stage is equally indispensable for the full length synthesis of HIV-1 mRNA. Several compounds targeting these stages have been studied in detail, some of which are currently in various stages of preclinical trials, including phorbol ester derivate prostratin [7], ingenol ester derivates PEP005 [8] and IngB [9, 10], hybrid polar compound HMBA (hexamethylene bisacetamide) [11–13], and BET bromodomain inhibitors BETi (JQ1, I-BET, I-BET151, and MS417) [14, 15]. The current challenge is to explore the use of these compounds in combination therapy so as to exploit their synergistic effect(s) in order to be used potently to reactivate latent HIV-1 transcription at the different stages without causing global T cell activation. Here, we tested the putative effects that combination of prostratin and HMBA had on latent HIV-1 transcription.

For almost a decade now, prostratin, a nontumorigenic phorbol ester, has been shown to promote transcriptional activation of latent HIV provirus [16, 17]. However, the precise molecular mechanism of prostratin's ability to activate HIV latency is still far from clear. Structurally, prostratin is similar to the well-studied phorbol ester PMA (phorbol-12-myristate-13-acetate), which can promote carcinogenesis via activation of the PKC pathway. Consistent with its structural features, prostratin has also been shown to activate the PKC pathway [18]. An elegant study of prostratin's effect in J-Lat T cell lines with integrated HIV proviruses has shown that, indeed, activation of the PKC pathway is critical for prostratin's stimulatory effect on the expression of latent HIV provirus [7].

HMBA, a prototype hybrid polar compound, was originally developed as a potent inducer of terminal differentiation of leukemia as well as solid tumor cell lines [19–22]. Our group and others have recently shown that HMBA could activate the positive transcription elongation factor P-TEFb [11] and its recruitment factor, bromodomain containing protein Brd4, thereby resulting in the activation of transcriptional elongation of HIV-1 [11–13]. However, the expression of HIV-1 not only does depend on transcription elongation, but also is contingent on NF-*κ*B-dependent transcription initiation [23]. In fact, a number of studies have demonstrated that HMBA could weakly stimulate the transcription initiation of HIV-1 through NF-*κ*B.

In unstimulated cells, NF-*κ*B associates with I*κ*B*α* and is thus retained in the cytoplasm. However, upon stimulation, the E3 ubiquitination enzymes induce the K63-linked polyubiquitination of upstream factors, such as RIP1, TRAF6, and IKK*γ*, which leads to the activation of the downstream kinase complex, IKK. The activated IKK in turn phosphorylates I*κ*B*α* for proteosomal degradation, thereby triggering the translocation of NF-*κ*B to the nucleus to induce the transcription of target genes, as well as negative regulators of NF-*κ*B signaling pathway I*κ*B*α* coupled with the expression of the deubiquitinases, A20 and CYLD [24]. Subsequently, A20 and CYLD induce the deubiquitination of K63-linked polyubiquitination of the upstream activators, which converts IKK back to its inactive state, thereby terminating the NF-*κ*B signaling [24]. Thus, the deubiquitinases A20 and CYLD play a central role in the negative feedback control of NF-*κ*B signaling pathway.

In this study, we report the synergistic effects of HMBA and prostratin cotreatment on the expression of latent HIV-1 provirus via HIV-1 transcription activation. While prostratin mainly enhances P-TEFb activation stimulated by HMBA, interestingly, HMBA boosts NF-*κ*B signaling activated by prostratin. We found that although HMBA alone had no effect on NF-*κ*B signaling, it enhanced and prolonged prostratin-induced nuclear translocation of NF-*κ*B by specifically suppressing the expression of A20, but not I*κ*B*α*. Therefore, by blocking the negative feedback circuit, HMBA functions as a signal amplifier in the NF-*κ*B signaling pathway. These findings might open new therapeutic strategies with drug combinations toward eradicating latent HIV-1 provirus.

## 2. Materials and Methods

### 2.1. Chemicals and Enzymes

Prostratin, Trichostatin A (TSA), and cyclosporin A are from Santa Cruz. Hexamethylene bisacetamide (HMBA) is from Sigma. MG-132 is from Boston Biochem. Bay 11-7082 is from Calbiochem. DyNAmo*™* ColorFlash Master Mix is from Thermo Scientific. M-MLV is from Takara. All other chemicals are from AMRESCO or Sigma.

### 2.2. Antibodies

Rabbit anti-A20 antibody is from Proteintech (Wuhan, China). Rabbit anti-RelA (ChIP grade), I*κ*B*α* antibodies are from Santa Cruz. Rat anti-HA antibody is from Roche. Mouse anti-Flag and *β*-Actin antibodies are from Sigma.

### 2.3. Plasmids

The ORF fragments of human A20 (TNFAIP3) (a gifts of Dr. JH Han, School of Life Sciences, XMU), NF-*κ*B/RelA [25] were subcloned into BamH I/Xba I sites of a modified pLV-Flag and pLV-HA lentiviral vector [12].

### 2.4. Cell Lines, Transfection, and Infection of Cells

HeLa cells, HEK293T cells, or HeLa cells with an integrated HIV-LTR luciferase reporter gene (HIV-LTR-Luc) were maintained as previously described [12]. Transfection and lentiviral infection were performed as previously described [11, 12]. The HIV latent cell lines were obtained from Verdin's laboratory [26].

### 2.5. Treatment of Cells with Various Pharmacological Compounds

Cells at 50% confluence were incubated with HMBA (H, 10 mM), prostratin (P, 2 *μ*M), TSA (T, 400 nM), or prostratin combined with HMBA or TSA for 4 or 6 hr. To observe the effects of various inhibitors on the corresponding signal pathways, cells were preincubated with Bay 11-7082 (Bay, 10 *μ*M) for 1 hr, followed by prostratin and/or HMBA treatment. The cell lysates were analyzed by Western Blot as previously described [12].

### 2.6. Luciferase Assay

Luciferase assay was performed with HIV-LTR-Luc integrated HeLa cells as previously described [12]. The error bars are standard deviations based on three independent experiments.

### 2.7. Chromatin Immunoprecipitation (ChIP)

ChIP was carried out with HIV-LTR-Luc integrated HeLa cells as previously described [12]. The immunoprecipitated DNA was analyzed by quantitative PCR with primers (forward: 5′-GCT GAT ATC GAG CTT GCT AC, reverse: 5′-CCA ACA GTA CCG GAA TGC C) in promoter region of HIV-1 construct. The results from two independent experiments were averaged and plotted as percentage of input.

### 2.8. Quantitative RT-PCR (qRT-PCR)

qRT-PCR was performed as previously described [12]. The gene expression levels were normalized to *β*-Actin and the error bars were calculated based on three independent experiments. The primers used are as follows: HIV-LTR-Luci (+1~+59, forward: 5′-GGG TCT CTC GAG TTA GAC CAG ATC TGA and reverse: 5′-GGG TTC CCT AGT TAG CCA GAG AGC); A20 (forward: 5′-ATT CAA GAT CGT TCT GTG G and reverse: 5′-GAA CCT CTC CTC ACA ATT GG); CYLD (forward: 5′-GAA CAA GTC CTC AGG and reverse: 5′-AAC TGG CAA GTT CTG AAC); I*κ*B*α* (forward: 5′-CCA GGG CTA TTC TCC CTA CC and reverse: 5′-GCT CGT CCT CTG TGA ACT CC); and *β*-Actin (forward: 5′-ATC GTC CAC CGC AAA TGC TTC T, reverse: 5′-AGC CAT GCC AAT CTC ATC TTG T).

### 2.9. Immunofluorescence (IF)

Transfected or untransfected HeLa cells were platted and cultured on glass coverslips and treated with the compounds indicated. After being washed with PBS, the cells were subjected to immunostaining and visualized with confocal microscopy as previously described [1, 11].

## 3. Results and Discussion

### 3.1. HMBA and Prostratin Synergistically Stimulate the Release of Latently Infected HIV-1 Provirus through Transcription Activation

**Figure 1:**
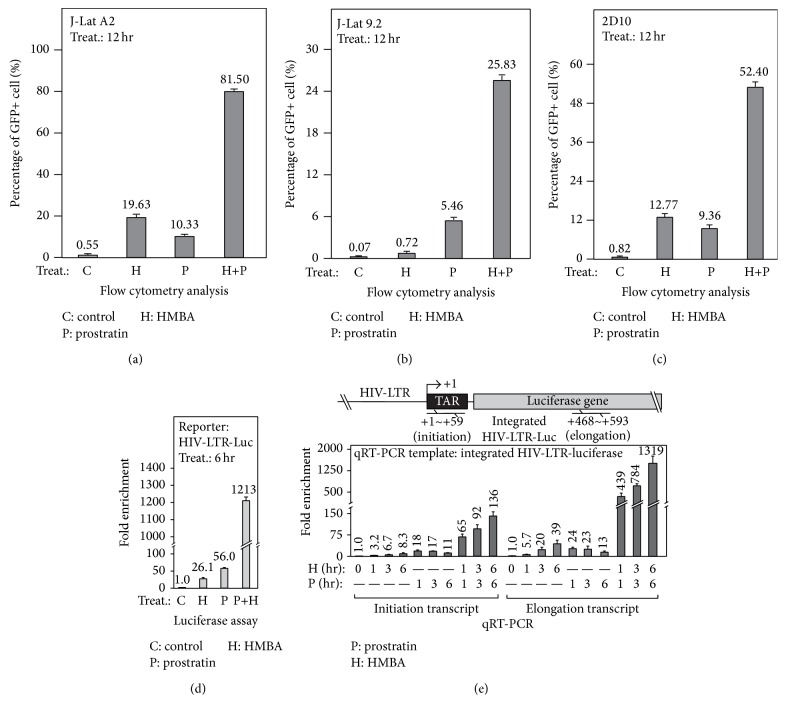
HMBA and prostratin synergistically antagonize HIV-1 latency in J-Lat T-lymphocytes by activating both transcriptional initiation and elongation. (a to c) HMBA and prostratin synergistically activate the replication of latent HIV-1 proviruses. J-Lat clones A2, 9.2, and 2D10 were treated with indicated pharmacological compounds. GFP expression was measured by flow cytometry. The percentage of GFP positive cells is presented to reflect the transcriptional activation of the latent HIV proviruses. (d) HMBA and prostratin synergistically activate HIV-LTR-Luc expression. HeLa cells with integrated HIV-LTR-luciferase reporter gene (HIV-LTR-Luc) were treated with indicated compounds. The cell lysates were prepared for luciferase assay for the expression of HIV-LTR-Luc. (e) HMBA and prostratin synergistically activate both transcriptional initiation and elongation of HIV-1. HIV-LTR-Luc cells were treated with indicated compounds. The isolated RNA was assayed by qRT-PCR for the initiation and elongation transcripts of HIV-LTR-Luc. The qRT-PCR fragments representing transcriptional initiation (+1~+59, TAR) and elongation (+468~+593, luciferase) are illustrated in top panel. For (d) and (e), data are presented as fold enhancement compared to untreated cells.

While prostratin has been shown to be capable of triggering NF-*κ*B-dependent transcriptional initiation of latently infected HIV-1 provirus [7], we and others have previously demonstrated that HMBA is capable of stimulating the transcriptional elongation of integrated HIV-1 [11–13]. To determine if the simultaneous activation of the initiation and elongation steps could synergistically enhance the release of latently infected HIV-1 provirus, J-Lat A2 cells, a popular latency model with truncated HIV-1 genome and HIV-LTR-driven eGFP [26] was used. Compared to cells treated with only prostratin or HMBA, we observed a marked increase in the number of GFP positive cells under cotreatment with prostratin and HMBA, as revealed by flow cytometry analysis (Figure 1(a)). Given that the HIV-1 genome in the J-Lat A2 cells is incomplete, we went on to employ two other typical HIV-1 latency models, that is, J-Lat 9.2 cells and 2D10 cells, which both harbor full length latent proviruses [26, 27]. Similarly, we observed a synergistic effect of these two compounds on the activation of provirus, as there was a marked increase in the number of GFP positive cells in cotreated cells as compared to cells treated with only prostratin or HMBA (Figures 1(b) and 1(c)).

To further explore the effect(s) of drug combination on HIV-1 activation, we then employed HeLa cells with an integrated HIV-LTR-luciferase reporter gene (HIV-LTR-Luc) [11, 28]. Intriguingly, cotreatment of these cells with prostratin and HMBA drastically increased luciferase activity more than 1000-fold, whereas treatment with only prostratin or HMBA resulted in only 26-fold and 56-fold increase in luciferase activity, respectively (Figure 1(d)). Apart from demonstrating the synergistic effects of these two compounds on HIV-1 activation, using qRT-PCR assay with specific primers targeting the initiation and elongation regions of the transcript (Figure 1(e), top) [12], we also confirmed our previous observation that prostratin mainly augmented transcriptional initiation, while HMBA mostly stimulated transcriptional elongation (Figure 1(e), bottom). Therefore, a combination of prostratin and HMBA synergistically activated the transcription of the HIV-1 reporter gene at both the initiation and elongation stages.

Interestingly, while HMBA alone or in combination with prostratin induced a time-dependent increase in transcription initiation and elongation, prostratin alone enhanced the transcriptional initiation of HIV-1 to 18-fold at 1 hr after treatment, which was reduced to 11-fold by 6 hr after treatment (Figure 1(e), bottom). This observation seems to suggest that because HMBA was able to strongly reverse the long-term decline of transcription initiation induced by prostratin, especially at 6 hr after treatment, it could imply HMBA was responsible for prolonging prostratin-activated transcription initiation.

### 3.2. Prostratin Amplifies HMBA-Stimulated HIV-1 Transcriptional Elongation by Augmenting HMBA-Induced P-TEFb Activation

**Figure 2:**
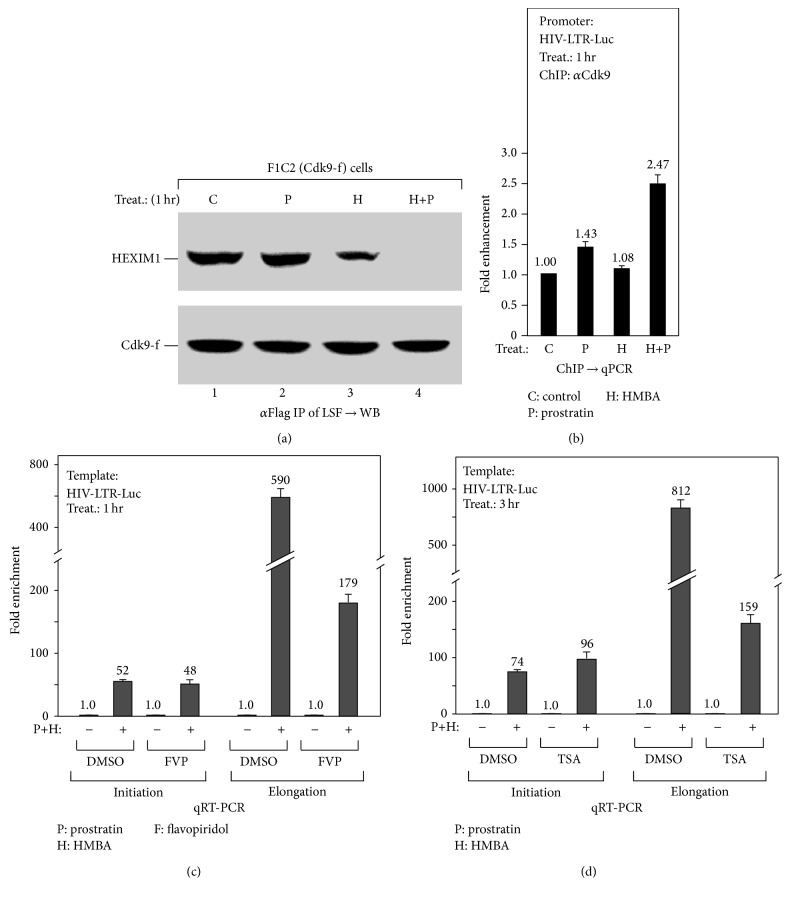
Prostratin enhances HMBA-stimulated HIV-1 transcriptional elongation by augmenting HMBA-induced P-TEFb activation. (a) Prostratin augments HMBA-induced P-TEFb activation. F1C2 (Cdk9-f) cells, a HeLa-based cell line stably expressing Cdk9-Flag, were treated as indicated, followed by low-salt extraction to yield low-salt faction (LSF). The anti-Flag immunoprecipitates of LSF were analyzed by Western Blot (WB) for the P-TEFb-bound HEXIM1 in LSF, which represents the level of inactive 7SK snRNP complex. (b) Prostratin enhances HMBA-induced P-TEFb recruitment on HIV-1 promoter. HIV-LTR-Luc cells with indicated treatment were assayed by anti-Cdk9 chromatin immunoprecipitation (ChIP) for the enrichment of P-TEFb on the promoter of HIV-LTR-Luc. Data from three independent experiments were averaged and presented as fold enhancement compared to untreated cells. (c and d) Inhibiting P-TEFb activation or recruitment only blocks H+P-induced transcriptional elongation of HIV-1. HIV-LTR-Luc cells were pretreated with flavopiridol (FVP) or histone deacetylase inhibitor TSA, followed by H+P treatment as indicated. The isolated RNA were analyzed by qRT-PCR for the initiation and elongation transcripts of HIV-LTR-Luc as in Figure 1(e).

Although prostratin together with HMBA synergistically increased HIV-1 transcription at both the initiation and elongation stages, the underlying mechanism is thought to be different. Our previous studies had revealed that HMBA could stimulate transcriptional elongation by releasing P-TEFb from the inactive 7SK snRNP complex, liberating Brd4 from chromatin, followed by the recruitment of active P-TEFb to the promoter by chromatin-free Brd4 [11, 12]. Thus, to investigate how prostratin enhanced HMBA-induced P-TEFb activation, we went about to fractionate F1C2 cells (stable CDK9-Flag based on HeLa cells) into LSF as previously described [11, 12]. This was then followed by the analysis of the level of P-TEFb-associated HEXIM1, as an indication of the amount of inactive 7SK snRNP complex, after anti-Flag affinity purification (Figure 2(a)), as well as the level of chromatin-free Brd4 (Figure S1(a) (see Supplementary Material available online at http://dx.doi.org/10.1155/2016/5173205)). As shown by the Western Blot analysis, prostratin augmented HMBA thereby triggering the disruption of inactive 7SK snRNP complex (Figure 2(a), lanes 3 and 4), but this did not affect or stimulate Brd4 liberation from chromatin (Figure S1(a), lanes 3 and 4). Consistently, active P-TEFb is shown to be enriched on the HIV-1 promoter, as demonstrated by the ChIP assay with anti-CDK9 antibody (Figure 2(b)). To further delineate the importance of P-TEFb activation and its recruitment in prostratin-enhanced HIV-1 transcription, the P-TEFb inhibitor, flavopiridol (FVP), and the histone deacetylase inhibitor, TSA (which inhibits Brd4 release), were used to block HIV-1 transcription following simulation by the drug combination (cotreatment). Interestingly, both FVP and TSA greatly impaired HIV-1 transcription at the elongation but not the initiation stage (Figures 2(c) and 2(d)). On the other hand, BAY 11-7082 (BAY), an IKK kinase inhibitor and an antagonist of NF-*κ*B signaling, was able to reduce HIV-1 transcription at both stages (Figure S1(b)). From the foregoing, it thus suggests that prostratin enhances HMBA-stimulated HIV-1 transcription by engaging in NF-*κ*B signaling so as to increase HMBA-activated transcriptional elongation. But most importantly, both P-TEFb activation and its recruitment by Brd4 were shown to be essential in this process.

### 3.3. HMBA Prolongs Prostratin-Induced NF-*κ*B Activation via I*κ*B*α* Degradation

**Figure 3:**
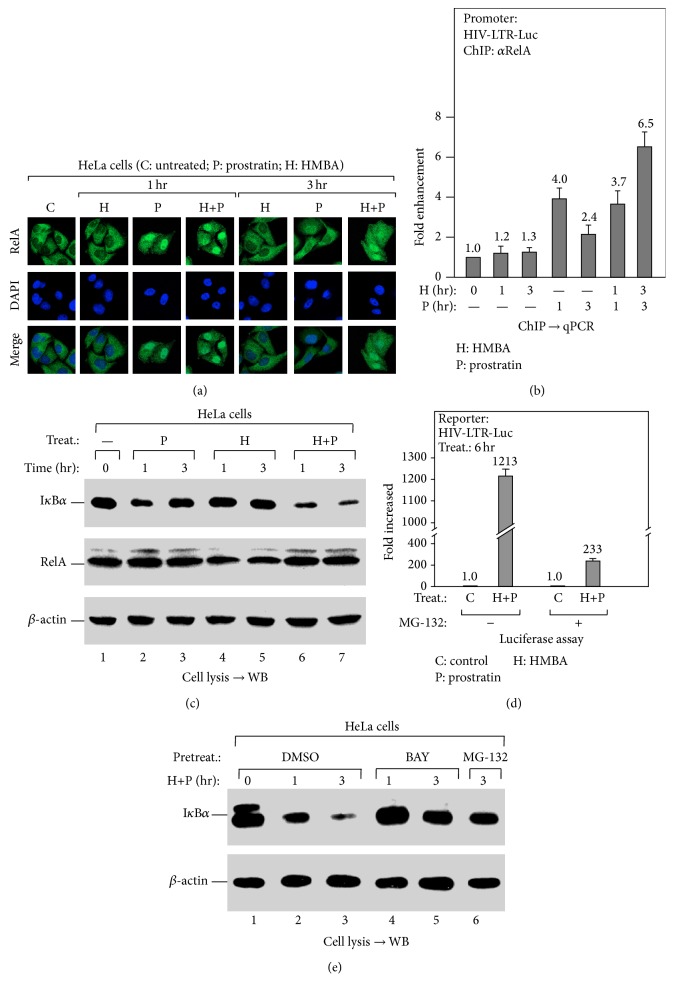
HMBA augments prostratin-activated NF-*κ*B by promoting prostratin-induced I*κ*B*α* degradation. (a) HMBA prolongs prostratin-induced nuclear translocation of RelA. HeLa cells with indicated treatments were assayed by anti-RelA immunofluorescence for the nuclear translocation of RelA. (b) HMBA enhances prostratin-activated recruitment of RelA on HIV-1 promoter. HIV-LTR-Luc cells with indicated treatments were assayed by anti-RelA chromatin immunoprecipitation (ChIP) for the enrichment of RelA on HIV-1 promoter as in Figure 2(b). (c) HMBA promotes prostratin-induced I*κ*B*α* degradation. The levels of I*κ*B*α* were analyzed by Western Blot (WB) using the cell lysates prepared from HeLa cells with indicated treatments. (d) Inhibiting I*κ*B*α* degradation by MG-132 blocks HMBA and prostratin cotreatment- (H+P-) activated HIV-1 expression. HIV-LTR-Luc cells with indicated pretreatment and treatment were subjected to luciferase assay for HIV-1 expression as in Figure 1(d). (e) Inhibiting IKK blocks H+P-induced I*κ*B*α* degradation. The levels of I*κ*B*α* were analyzed by WB using the cell lysates derived from HeLa cells with IKK inhibitor BAY preincubation, followed by indicated treatments.

Once we were able to establish that prostratin augments HMBA in HIV-1 transcriptional activation, we then went about to explore the distinct mechanisms by which HMBA contributes to prostratin-induced HIV-1 transcription. Thus, given that prostratin had been reported to activate HIV-1 transcription via NF-*κ*B signaling [23], we went on to examine the localization of NF-*κ*B following drug (prostratin and HMBA) treatment, using immunofluorescence (IF) assay. We observed that, in addition to its effect on transcription initiation (Figure 1(c)), prostratin also induced nuclear translocation of NF-*κ*B at 1 hr after drug treatment, followed by cytoplasmic export by 3 hr after treatment (Figure 3(a)). Notably, although HMBA did not alter the localization of NF-*κ*B, it did prolong prostratin-induced nuclear translocation of NF-*κ*B even at 3 hr after drug treatment (Figure 3(a)). Consistent with these observations, a chromatin immunoprecipitation (ChIP) assay also revealed that the promoter binding of NF-*κ*B/RelA was prolonged in cells cotreated with HMBA and prostratin (Figure 3(b)). This data therefore suggests that HMBA enhances prostratin-activated transcription initiation by prolonging prostratin-induced NF-*κ*B activation.

Given that HMBA was able to prolong prostratin-induced nuclear translocation of NF-*κ*B, we went further to determine the effect(s) of HMBA and/or prostratin treatment on the expression of I*κ*B*α*, a protein capable of blocking NF-*κ*B's nuclear translocation. Consistent with the observation of NF-*κ*B translocation when the cells were treated with only prostratin, the level of I*κ*B*α* was reduced at 1 hr after drug treatment but recovered to the original level by 3 hr after treatment (Figure 3(c), lanes 2 and 3). Interestingly, although HMBA alone did not affect the level of I*κ*B*α* (Figure 3(c), lanes 4 and 5), HMBA and prostratin cotreatment persistently reduced I*κ*B*α* level even at 3 hr after treatment (lanes 6 and 7), implying that HMBA could enhance prostratin-induced reduction of I*κ*B*α*.

To determine if the enhanced reduction in the level of I*κ*B*α* protein was due to its degradation or inhibition in its synthesis, we employed the use of the proteasome inhibitor, MG-132. As shown in Figure S2, pretreatment of cells with MG-132 resulted in the accumulation of I*κ*B*α*, especially in the cells treated with prostratin or prostratin plus HMBA, but not in those treated with only HMBA. This observation thus suggests that the reduction in the level of I*κ*B*α* is due to the stimulation-induced degradation, but not due to the inhibition of its synthesis. Similarly, we observed that HIV-1 expression following stimulation by prostratin and HMBA cotreatment was equally impaired by MG-132 pretreatment, as indicated by luciferase analysis (Figure 3(d)).

IKK kinase-mediated phosphorylation of I*κ*B*α* is a prerequisite for its degradation [24]. Thus, to determine if the kinase activity of IKK was involved in inducing I*κ*B*α* degradation following HMBA and prostratin cotreatment, cells were pretreated with the IKK inhibitor, Bay 11-7082 (BAY), and analyzed by Western Blot. As shown in Figure 3(e), indeed, inhibition of the IKK kinase did abolish the degradation of I*κ*B*α* (Figure 3(e)). This data is consistent with the notion that HMBA is capable of prolonging prostratin-induced IKK kinase activity so as to phosphorylate I*κ*B*α* for degradation, thereby enhancing NF-*κ*B activation.

### 3.4. HMBA Attenuates Prostratin-Induced Expression of A20

**Figure 4:**
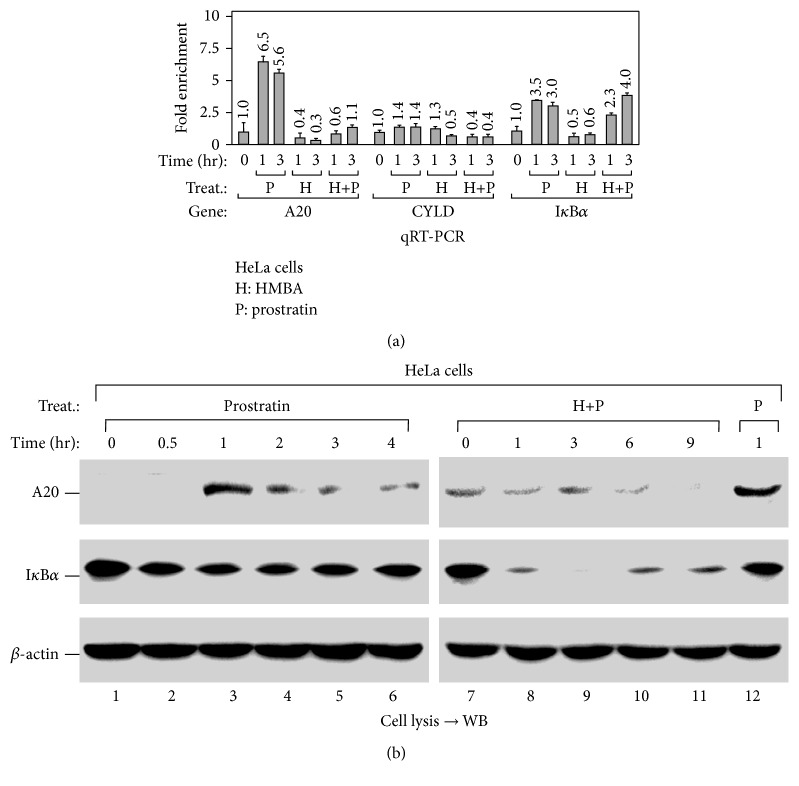
HMBA suppresses prostratin-induced A20 expression. (a) The mRNA levels of indicated genes were analyzed by qRT-PCR using total RNA isolated from the HeLa cells with indicated treatments. Data were presented as fold enhancement compared to untreated cells. (b) The A20 protein levels of the HeLa cells with indicated treatments were analyzed by Western Blot (WB).

The NF-*κ*B signaling pathway is regulated by multiple negative feedback factors, including I*κ*B*α* and the deubiquitinases, A20 and CYLD [24]. To determine how HMBA enhances prostratin-induced NF-*κ*B activation, we went about to examine the mRNA levels of A20, CYLD, and I*κ*B*α* following prostratin and/or HMBA treatment using qRT-PCR. Interestingly, while prostratin alone was able to enhance the expression of both A20 and I*κ*B*α*, the addition of HMBA resulted in inhibition of prostratin-induced expression of A20, but not I*κ*B*α* (Figure 4(a)). Consistent with the level of the mRNA expression (Figure 4(a)), we also observed that while prostratin-induced an increase in the level of A20 protein, this was abolished by the addition of HMBA (Figure 4(b)). Collectively, this data thus suggests that the enhancing effect of HMBA on the activation of NF-*κ*B is mostly likely due to its suppressive effect on prostratin-induced expression of A20.

### 3.5. Overexpression of A20 Abolishes HMBA's Ability to Enhance NF-*κ*B Activation

**Figure 5:**
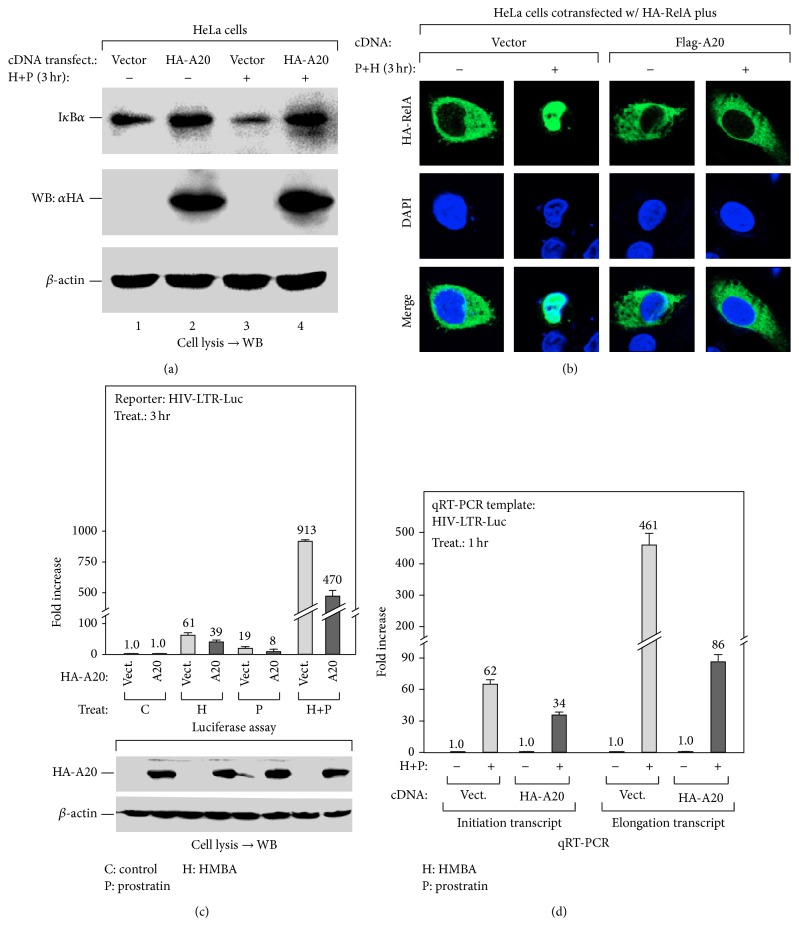
Overexpressing A20 abolishes HMBA's enhancing effect on prostratin-induced NF-*κ*B activation. (a) A20 overexpression attenuates HMBA-enhanced I*κ*B*α* degradation which was induced by prostratin. HeLa cells infected with lentivirus expressing A20 were treated as indicated. The levels of I*κ*B*α* in cell lysates were analyzed by Western Blot (WB). (b) A20 overexpression abolishes HMBA and prostratin- (H+P-) activated nuclear translocation of RelA. HeLa cells cotransfected with HA-RelA plus empty vector or Flag-A20 constructs were treated as indicated, followed by immunofluorescence analysis with anti-HA antibody. (c) A20 overexpression inhibits H+P-activated HIV-1 expression. HIV-LTR-Luc cells transfected with HA-A20 construct or empty vector were treated as indicated. The cell lysates were prepared for luciferase assay as in Figure 1(d). (d) A20 overexpression attenuates H+P-activated HIV-1 transcriptional initiation and elongation. HIV-LTR-Luc cells transfected with HA-A20 construct or empty vector were treated as indicated. The RNA was isolated for qRT-PCR analysis as in Figure 1(e).

If HMBA is capable of enhancing prostratin-induced NF-*κ*B activation by suppressing A20 expression, then it is expected that the overexpression of A20 should abolish this effect. Indeed, we observed a block in I*κ*B*α* degradation in HeLa cells transiently overexpressing A20 following cotreatment with HMBA and prostratin (Figure 5(a)). Moreover, in cells ectopically expressing A20, the nuclear translocation of HA-RelA, which is induced following HMBA and prostratin cotreatment, was no longer observed (Figure 5(b)). Intriguingly, the ectopic expression of A20 also inhibited the expression of the HIV-1 reporter gene induced following cotreatment with HMBA and prostratin (Figure 5(c)). But most importantly, the inhibition of HIV-1 expression following HMBA and prostratin cotreatment occurred at both the transcriptional initiation and elongation stages (Figure 5(d)).

Taken together, the above data have so far revealed a mechanism whereby suppressing the expression of A20 by HMBA increases prostratin-induced activation of the NF-*κ*B signaling pathway thereby enhancing the transcription initiation of integrated HIV-1 genes.

## 4. Conclusions

In this present study, we unexpectedly found that HMBA exerts a strong stimulatory effect on prostratin-induced transcription initiation thereby enhancing the process (Figure 1). Probing further, we found that prostratin enhanced HMBA-induced HIV-1 transcription by improving the stimulation of P-TEFb activation (Figure 2). Similarly, we noticed that HMBA could amplify prostratin-induced NF-*κ*B activation by enhancing the phosphorylation and degradation of I*κ*B*α* (Figure 3). Interestingly, the ability of HMBA to exert an amplifying effect is achieved by suppressing the expression of the negative feedback regulator, A20, but not I*κ*B*α* (Figure 4). Collectively, the results thus far have revealed the underlying mechanisms whereby a combination of compounds (drugs) synergistically increases HIV-1 transcription. It further revealed something novel, wherein HMBA is capable of breaking the negative feedback circuit of the NF-*κ*B signaling pathway by inhibiting the expression of the deubiquitinase, A20, therefore enhancing prostratin-induced NF-*κ*B activation (Figure 5).

In accordance with this study, a positive effect of HMBA on the activation of the NF-*κ*B signaling pathway in cytomegalovirus infection has previously been reported. Although, in that study, the molecular mechanisms could not be delineated, the ability of HMBA to enhance I*κ*B*α* degradation and NF-*κ*B translocation was also observed [23]. Similarly, HMBA has been reported to repress TNF-*α*-activated NF-*κ*B signaling by inhibiting TNF-*α*-induced activation of Akt and MEK [29]. This observation seems to suggest that probably not all stimuli could activate the expression of A20 or that HMBA is unable to suppress A20 expression following stimulation by some other stimuli. Consistent with this premise, our results so far indicate that although both I*κ*B*α* and A20 are NF-*κ*B target genes and their expression was induced by prostratin treatment, a combination with HMBA produced distinct effects on their expression (Figure 4(a)). A similar observation has previously been reported by Dey and colleagues, where HMBA was shown to attenuate TNF-*α*-induced expression of ICAM-1 and MCP-1 but had little or no effect on FLIP and BCLxL, all of which are NF-*κ*B-dependent genes [29]. It is therefore important to carefully consider all of these factors when planning combination treatment regimens.

In pharmacological research, one of the commonly used drug screening strategies is to first screen substances for their individual efficacy or potency before the suitable hits are combined. Here, we report of an intriguing finding where HMBA alone is unable to activate NF-*κ*B but was able to dramatically enhance prostratin-induced NF-*κ*B activation and further synergistically activate HIV-1 latency. There are several similar reports that have focused on in-depth screening of potential compound combinations for releasing HIV-1-latency in resting CD4+ T cells from patients. Interestingly, the high effective reactivation of HIV-1 latency always came through stimulation of the PKC-NF-*κ*B signaling pathway together with modulating P-TEFb activity [30]. Till date, three groups of PKC activators have been tested for reversing HIV-1 latency. These are phorbol esters derivatives including PMA, prostratin, and DPP, diterpene esters including PEP005 or IngB, and bryostatin-1. For P-TEFb modulators, other than HMBA, the most important agent is JQ1, which specifically antagonized Brd4 inhibition toward Tat-transactivation in cells with latent HIV-1. Other groups have also shown that HDAC inhibitors, such as SAHA, TSA, and VPA, were agents capable of reactivating HIV-1 transcription from latency [30]. However, we have previously shown that HDACs benefited Brd4 activation through histone deacetylation leading to chromatin release [13]. Therefore, these HDAC inhibitors also enhanced Tat-transactivation of latent HIV-1 by blocking Brd4 activation.

Nevertheless, while this observation is interesting and novel, further future analysis employing high-throughput techniques, humanized mice models, and CD4+ T cells from patients would enable unbiased testing of these compound combinations or their analogues so as to discover new therapeutic options for HIV-1 and other related infections.

## Supplementary Material

Prostratin did not further stimulate HMBA-induced Brd4 liberation from chromatin. Antagonizing the NF-κB signaling by BAY, an IKK inhibitor, could block HMBA and prostratin co-treatment (H+P)-induced transcriptional activation of HIV-LTR-Luc at both the initiation and elongation stages.
HMBA could enhance prostratin-induced degradation of IκBα, while MG-132 pre-treatment could block this stimulation-induced degradation.
