# Supplementary material for: HMBA Enhances Prostratin-Induced Activation of Latent HIV-1 via Suppressing the Expression of Negative Feedback Regulator A20/TNFAIP3 in NF-κB Signaling
Source: Biomed Res Int. 2016 Jul 27;2016:5173205. doi: 10.1155/2016/5173205 (PMC4978819; doi:10.1155/2016/5173205)
Supplement: Supplementary file 1 — Prostratin did not further stimulate HMBA-induced Brd4 liberation from chromatin. Antagonizing the NF-κB signaling by BAY, an IKK inhibitor, could block HMBA and prostratin co-treatment (H+P)-induced transcriptional activation of HIV-LTR-Luc at both the initiation and elongation stages. HMBA could enhance prostratin-induced degradation of IκBα, while MG-132 pre-treatment could block this stimulation-induced degradation. [file 5173205.f1.pdf]

Supplemental figure legends

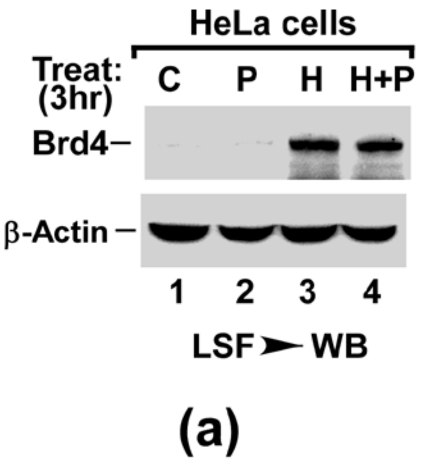

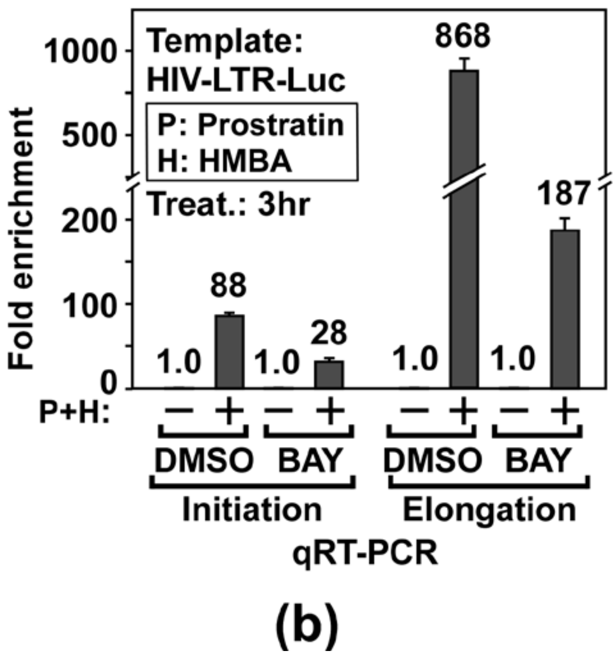

FIGURE S1. Prostratin induces the transcriptional initiation via NF-κB signal pathway. (a) Prostratin does not induce Brd4 activation. HeLa cells expressing f-RelA were treated as indicated, followed by low-salt fraction (LSF) preparation. The LSF was assayed by Western blot (WB) for the levels of free Brd4. **(b)** Inhibiting IKK by BAY blocks HMBA and prostratin co-treatment (H+P)-induced transcriptional initiation and elongation of HIV-LTR-Luc. HIV-LTR-Luc cells were pre-treated with BAY for 1 hr, followed by H+P treatment as indicated for 3 hr. The isolated RNA was assayed by qRT-PCR for the initiation and elongation transcripts of HIV-LTR-Luc as in Fig. 1E. BAY: IKK inhibitor BAY 11-7082.

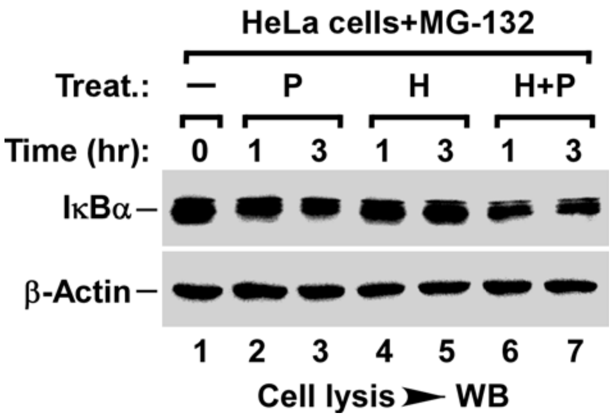

FIGURE S2. MG-132 blocks HMBA and prostratin-induced IκBα degradation. Levels of IκBα were analyzed by Western blot (WB) using the cell lysates prepared from HeLa cells with indicated pre-treatment and treatments. MG-132: proteasome inhibitor.
